# Facile Synthesis of Nanoporous NiS Film with Inverse Opal Structure as Efficient Counter Electrode for DSSCs

**DOI:** 10.3390/ma13204647

**Published:** 2020-10-18

**Authors:** Xu Chen, Yang Zhang, Yashuai Pang, Qiwei Jiang

**Affiliations:** 1 Department of Physics, School of Physics and Electronic, Henan University, Kaifeng 475004, China; chenxu-qaz@vip.henu.edu.cn (X.C.); yzhang@henu.edu.cn (Y.Z.); pangyashuai@vip.henu.edu.cn (Y.P.); 2 Institute of Macro/Nano Photonic Materials and Application, Henan University, Kaifeng 475004, China

**Keywords:** electrocatalytic activity, nanoporous NiS film, template method, dye-sensitized solar cells, counter electrode

## Abstract

To satisfy the high requirement of catalytic activity for efficient dye-sensitized solar cells (DSSCs), a novel nanoporous NiS film with inverse opal structure and outstanding electrocatalytic properties was prepared by a facile template-assisted electrodeposition method. The inverse opal structure makes the film have a larger specific surface area and more catalytic sites, thereby result to a higher electrocatalytic activity. Compared with the flat NiS/FTO electrode, this kind of nanoporous NiS film with inverse opal structure has higher catalytic activity and can be used as a cheap and efficient Pt-free electrode to replace the traditional Pt/FTO electrode. It is of great significance to reduce the cost and promote the wide application of DSSCs. This study opens up a new experimental exploration for further improving the catalytic activity of NiS electrode and the according photovoltaic efficiency of DSSCs. The template-assisted electrodeposition method proposed in this work provides a facile method for morphology control and an easy to be realized way to optimize the catalytic performance of the metal sulfides counter electrode.

## 1. Introduction

Dye-sensitized solar cells (DSSCs) have shown promise as low-cost photovoltaics compared to commercially available Si solar cells [1,2,3]. The standard DSSC consists of a dye-sensitized mesoscopic TiO_2_ photoanode, a platinized counter electrode (CE), and an electrolyte containing a redox couple [4]. As a key component, CE plays the role of collecting electrons from external circuit and promoting the regeneration of redox couple [5]. This essential function needs the CE possessing excellent electrocatalytic activity; the traditional platinized FTO (fluorine-doped tin oxide) electrode (Pt/FTO) can meet this requirement and can be used as efficient CE for DSSCs. However, the platinum (Pt) is a rare metal and its price is high [6]. So, it is highly important to develop new low-cost Pt free CEs with high electrocatalytic activity.

**Figure 1:**
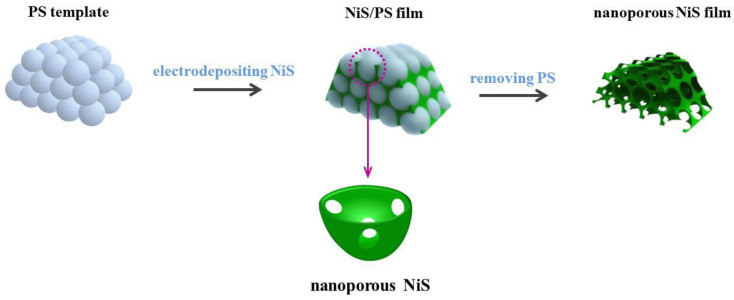
Schematic diagrams of the preparation process and structure of nanoporous NiS film.

Up to now, various materials, including carbon-based materials [7,8,9], transition metal compounds [10,11], conducting polymers [12], and their composites have been extensively studied as cost-effective substitutes for Pt [13]. Among these materials, the NiS is particularly attractive, due to its excellent electrocatalytic activity and outstanding electrochemical stability [14]. A series of NiS materials with different morphology, including NiS nanowall networks [15], NiS/Ni_3_S_2_ nanorod composite array [16], sphere-like NiS [17], NiS nanoparticles-decorated graphene [18], NiS nanorice [19], NiS cubes [20], and NiS nanosheets, were successfully synthesized via the hydrothermal method. Furthermore, the electrodepositing of NiS layer on conductive substrate is proved an easy to be realized method for preparing efficient NiS CEs [21]. However, the surface of NiS film electrodeposited on FTO by this method is usually flat. Although this kind of NiS film has good catalytic activity, the flat NiS surface limits the specific surface area and the number of catalytic sites. The limited catalytic sites will hinder the catalytic activity of CE. The CE with excellent catalytic activity is beneficial to the further improvement of the photovoltaic efficiency, this also means that DSSCs with higher photoelectric efficiency requires the CE to have outstanding catalytic activity. It is generally believed that compared with flat film, the porous structure can let CE own a larger surface area and more catalytic sites, resulting to a higher catalytic activity [16]. In addition, the porous structure of CE is also beneficial to the diffusion of redox pairs (I_3_^−^/I^−^) between electrodes, which is also in favor of the regeneration of redox pairs and the overall photovoltaic efficiency of DSSCs [22]. Therefore, to satisfy the high requirement of electrocatalytic activity for efficient DSSCs, it is of great significance to let the electrodeposited NiS have a porous structure. The porous films with inverse opal structure are attractive materials for various applications in electrochemical devices because of the benefits derived from the structures: relatively large surface areas, large voidage, and interconnected macropores [23,24,25]. A series of materials with inverse opal structure were successfully prepared on polystyrene microspheres (PS) template by electrodeposition [26,27,28,29,30]. Inspired by this, herein, we design a kind of nanoporous NiS film with inverse opal structure and satisfactory electrocatalytic performance, which was prepared by a facile template-assisted electrodeposition method. The PS film was used as templates, a layer of NiS was electrodeposited on its surface, and the nanoporous NiS film was obtained after removing the PS template (Figure 1). The properties of this kind of CE with inverse opal structure were systematically studied in this work. The results show that the PS template-assisted electrodeposition is a facile method to compose the nanoporous NiS film with excellent electrocatalytic activity.

## 2. Experimental Section

### 2.1. Materials and Reagents

Styrene (C_8_H_8_), potassium persulfate (K_2_O_8_S_2_), nickel sulfate hexahydrate (NiSO_4_^.^6H_2_O), thiourea (CH_4_N_2_S), boric acid (H_3_BO_3_), sodium hydroxide (NaOH), potassium chloride (KCl), chloroplatinic acid hexahydrate (H_2_PtCl_6_^.^6H_2_O), titanium(IV) tetrachloride (TiCl_4_), polyethylene glycol (PEG-20000), acetonitrile (C_2_H_3_N), ethanol (C_2_H_6_O), 1,2-dimethyl-3-propylimidazolium iodide (C_8_H_15_IN_2_), iodine (I_2_), 4-tert-butyl-pyridine (C_9_H_13_N), guanidinium thiocyanate (CH_5_N_3_^.^CHNS), nitric acid (HNO_3_), and lithium iodide (LiI) were purchased from Aladdin (Shanghai, China). Titanium isopropanol (Ti{OCH(CH_3_)_2_}_4_) and N,N-dimethylformamide (C_3_H_7_NO) were purchased from Sigma-Aldrich (USA). Dye N719 (Ruthenium 535 bis-TBA) was purchased from Solaronix (Aubonne, Switzerland). FTO glass (15 Ω/sq) was purchased from Nippon Sheet Glass (Tokyo, Japan). Adhesive tape (Scotch Magic Tape 810) was purchased from 3M (St Paul, MN, USA). All agents are analytical grade and used without any purification.

### 2.2. Preparation of PS Suspension

The polystyrene microspheres (PS) were composed by polymerizing styrene (C_8_H_8_) monomer in the water solution [31]. First, 30 mL of styrene monomer was added into a separatory funnel containing 150 mL of aqueous solution and 0.1 mol/L NaOH, and the mixture was sufficiently shaken and washed for 3 times to remove polymerization inhibitor therein. Then, deionized water was added and washed for several times until the washing liquid was neutral. Then, 30 mL of washed styrene monomer and 180 mL of deionized water were added into a three-necked flask with a condenser tube and a thermometer and placed in a constant temperature magnetic stirrer under the protection of flowing argon, and magnetic stirring was maintained at a constant temperature water bath at 70 °C. Then, 0.2 g of potassium persulfate was dissolved in 20 mL of deionized water, and after full dissolution, the solution was slowly dripped into the three-necked flask (dripping was completed within 10 min) to initiate the polymerization reaction, which was continued to react for 6 h under the protection of flowing argon. Afterwards, the obtained milky white suspension of PS was cooled to room temperature, centrifuged, and washed, and then, it was stored in deionized water for later use.

### 2.3. Preparation of PS Template Film

The PS template film was prepared on FTO conductive glass substrate by the pulling method. The FTO conductive glass was washed with deionized water and absolute ethyl alcohol, respectively, dried and vertically immersed in the PS dispersion, stood for 3 min, and then, pulled up at a rate of 5 cm/min to make a layer of PS adhere to the conductive surface of FTO. After pulling, it was dried at 40 °C for 10 min. Then, the FTO conductive glass attached with a layer of PS was vertically immersed in the PS dispersion liquid again and pulled at the same rate. As a result, another layer of PS was attached on FTO substrate. The above process was repeated several times to obtain the PS template film on the FTO conductive glass substrate. The thickness of the film can be controlled through the different numbers of the pulling. In order to make PS adhere only to the conductive side of FTO, a layer of adhesive tape was covered on the insulating side of FTO during the preparation of PS template film. After the PS template was prepared, the tape was torn off. The dipping and pulling of the FTO substrate into the PS suspension were repeated for 3 times; as a result, the PS film with a thickness of 6 μm was obtained.

### 2.4. Preparation of Nanoporous NiS Electrode

Under strong stirring, 5.62 g NiSO_4_^.^5H_2_O and 3 g thiourea were dissolved in 30 mL deionized water. Briefly, 1.2 g H_3_BO_3_ was added in the solution to stabilize the pH, 0.6 g of KCl was added to improve the conductivity, and 8 mL of anhydrous isopropanol was added in the solution to reduce the surface tension of the aqueous solution, thus obtaining the plating solution for electrodepositing NiS. Using PS template film on FTO (PS/FTO) as cathode and metal Ni sheet as anode, the NiS electrodeposition was carried out by an electrochemical workstation with periodic reverse pulse potential method [21]. The NiS was electrodeposited on PS template film at −0.9 V potential for 3 s and then treated with 2 V reverse potential for 10 s; this forms one deposition cycle. After repeating the deposition for 20 cycles, a layer of NiS shell was coated on the outer layer of PS, and so the NiS/PS composite film coated with NiS was obtained. The thickness of NiS was controlled by the number of electrodeposition cycles. Then, the obtained NiS/PS film was washed for three times in deionized water, and after drying, it was immersed in a polytetrafluoroethylene reactor containing N,N-dimethylformamide and heated at 120 °C for 30 min to remove the PS template. After cooling to room temperature, obtained NiS/PS film was washed with anhydrous ethanol. Thus, the nanoporous NiS film was obtained on FTO substrate.

### 2.5. Preparation of Flat NiS/FTO Electrode and Flat Pt/FTO Electrode

The flat NiS/FTO electrode and Pt/FTO electrode were prepared based on FTO conductive glass. The NiS was electrodeposited on FTO substrate to form flat NiS/FTO electrode; the electroplating solution and the electrodeposition method were similar with that of the nanoporous NiS film, and the deposition time lasted for four deposition cycles. The Pt/FTO electrode was obtained by electrodepositing a Pt layer on the surface of FTO in the plating solution containing H_2_PtCl_6_^.^6H_2_O (5 g/L) under the constant potential of −2.8 V [32].

### 2.6. Assembly of DSSC

The dye-sensitized TiO_2_ electrode was prepared with the following process [33]. For this, 20 mL titanium isopropanol in 120 mL of 0.1 mol/L nitric acid aqueous solution was added under violent stirring. After aging at 80 °C for 8 h, the solution turns to a semitransparent blue white colloidal suspension. The obtained TiO_2_ colloidal suspension was heated in a polytetrafluoroethylene reactor at 200 °C for 12 h. Then, the obtained TiO_2_ slurry was concentrated and 1.2 g PEG-20000 was added to form the TiO_2_ colloid. The TiO_2_ colloid was coated on FTO by the doctor blading method to form the TiO_2_ film. After sintering in muffle furnace at 450 °C for 30 min, the TiO_2_ film was immersed in 40 mmol/L TiCl_4_ aqueous solution at 70 °C for 30 min, and then sintered in muffle furnace at 450 °C for 30 min. After cooling to room temperature, the prepared TiO_2_ electrode (film thickness of 12 μm) was immersed in the dye N719 ethanol solution (0.3 mM) in the absence of light for 12 h to adsorb the dye. The electrolyte used in this study was a liquid mixture consisted of 0.10 M of LiI, 0.03 M I_2_, 0.30 M 1,2-dimethyl-3-propylimidazolium iodide, 0.50 M 4-tert-butyl pyridine, and 0.10 M guanidinium thiocyanate in acetonitrile solution.

### 2.7. Characterizations

The morphology of the samples and the film thickness were detected using scanning electron microscopy (SEM, Joel 7000F, Tokyo, Japan) equipped with an energy-dispersive X-ray spectrometer (EDS). The DSSCs were illuminated by a solar simulator (Trusttech CHF-XM500, Beijing, China) under the radiation of 100 mWcm^−2^ irradiation. The photocurrent-voltage (J-V) characteristic curves of DSSCs were recorded using RST-5200D electrochemical workstation (Shiruisi, Zhengzhou, China). The Tafel curves and the electrochemical impedance spectroscopy (EIS) of the electrodes were measured using the same electrochemical workstation. The EIS in the symmetric cell configuration with two identical CEs (the electrolyte used is the same as that used in DSSCs) were measured with an AC modulation signal of 20 mV and a bias voltage of 0.3 V, with the frequency range from 100 kHz to 100 mHz.

## 3. Results and Discussion

**Figure 2:**
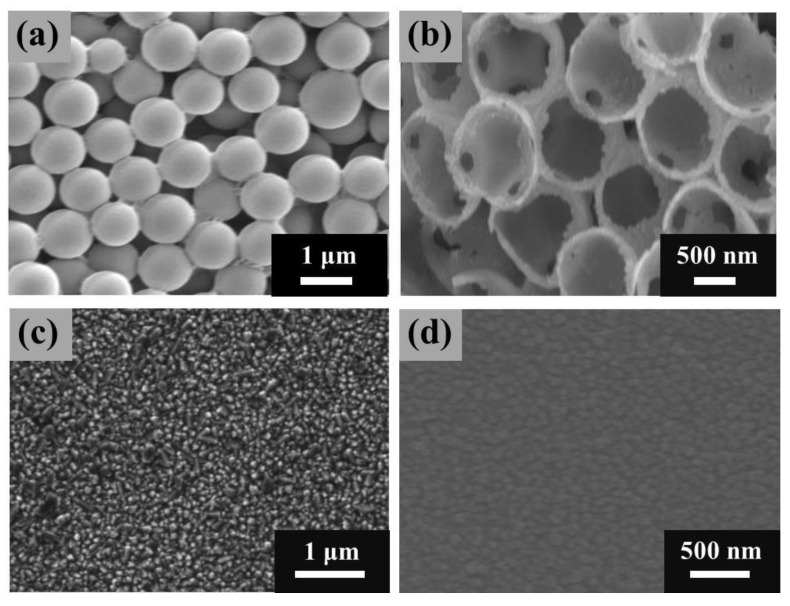
SEM images of the polystyrene microspheres (PS) template film (**a**), nanoporous NiS film (**b**), flat Pt/FTO (**c**), and flat NiS/FTO (**d**).

The SEM of the synthesized PS template film is shown in Figure 2a, the polystyrene microspheres are uniform in size, regular in shape, and smooth in surface. The diameter of the microspheres is about 1 μm, and the gap space between the microspheres is sufficient to meet the needs of NiS deposition. By the facile electrodeposition, a layer of NiS was electrodeposited on the PS template film. As a result, the PS was uniformly coated with a NiS shell. The growth process of NiS/PS composite film was studied by observing the cross-section of NiS/PS composite film with scanning electron microscope. It was found that the electrodeposited NiS layer is dense, and it grows from the surface of the conductive FTO substrate and then gradually covers the surface of PS template film from bottom to top. After removing the PS template, the nanoporous NiS film was formed. The thickness of the NiS film was controlled by the electrodepositing time. It was found that after repeating the NiS electrodeposition for 20 cycles, a NiS layer with the thickness of 3 μm grown from FTO can be obtained. It can be clearly seen from Figure 2b that the connecting holes are formed after the PS is dissolved. The formed nanoporous NiS film show an inverse opal structure, and the electrodeposited NiS shell (the shell thickness is 50–70 nm) on the PS template form a tightly connected inverse opal skeletons, the formed pore diameter is about 150–200 nm. The specific surface area of the nanoporous NiS film (the film thickness is about 3 μm) with inverse opal structure is obviously higher than that of the traditional flat Pt/FTO (Figure 2c) film and flat NiS/FTO film (Figure 2d), this means that the contact area between the nanoporous NiS film and the electrolyte is larger, which will provide more catalytic sites for the I_3_^−^ electron receiving reaction and promote the transfer of electrons at the CE/electrolyte interface. The surface area can be estimated by the double-layer capacitance of CEs (Table S1). Compared with the flat NiS/FTO film, the capacitance of NiS is much larger, indicating that it has a larger specific surface area. Moreover, the porous structure is benefit to the electrolyte (I_3_^−^/I^−^) diffusion, enabling DSSCs obtain higher photovoltaic efficiency.

**Figure 3:**
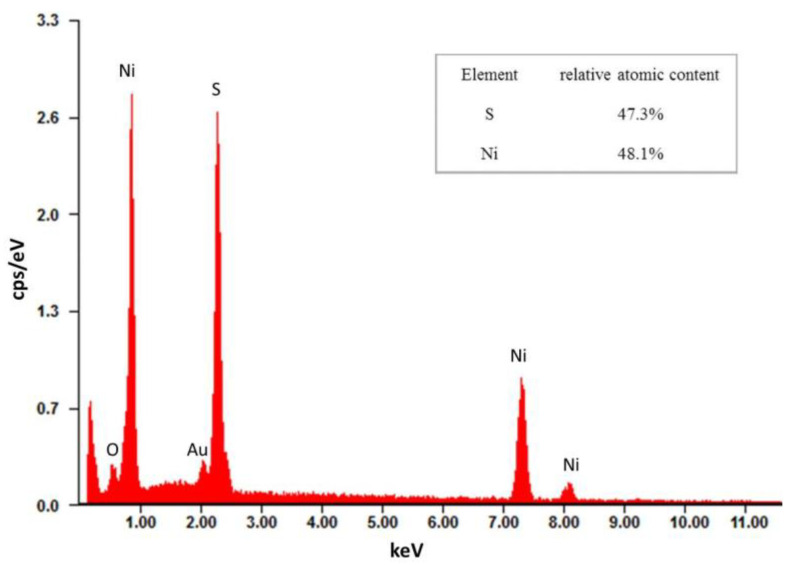
EDS spectrum of nanoporous NiS film.

The EDS spectrum (Figure 3) shows that the relative atomic content of S and Ni in the sample nanoporous NiS film is 47.3% and 48.1%, respectively, and the atomic ratio of S to Ni is close to 1:1, indicating that the electrodeposition obtained is NiS.

**Figure 4:**
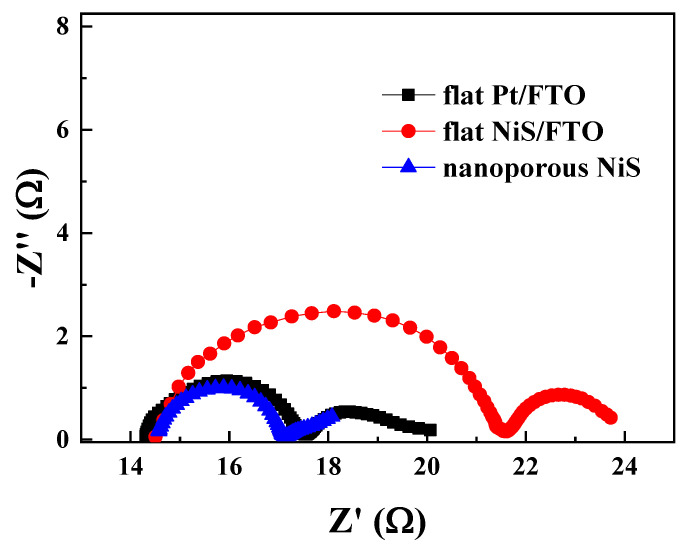
Nyquist plots of nanoporous NiS, flat NiS/FTO, and flat Pt/FTO.

The EIS of different CEs were measured in a symmetric cells (CE–CE) system. The equivalent circuit for fitting electrochemical impedance spectroscopy (EIS) plots was shown in Figure S1. As shown in Figure 4, each Nyquist plot is composed of two semicircles, the semicircle in the high frequency region represents the impedance corresponding to the charge transfer between CE and electrolyte (R_ct_) [34]. The impedance of the low frequency region corresponds to the Nernst diffusion impedance (Z_W_) of electrolyte. Generally, the smaller the R_ct_ value, the better the electrocatalytic performance of the CE [35]. The sheet resistance (R_s_) values of each CE are as follows: flat Pt/FTO (14.53 Ω) < nanoporous NiS (14.59 Ω) < flat NiS/FTO (14.60 Ω). Due to the excellent conductivity of Pt/FTO, its R_s_ value is slightly smaller than that of NiS CE, but the difference is very small, indicating that the deposited NiS has excellent conductivity, and the R_s_ values of nanoporous NiS electrode and flat NiS/FTO electrode have no much difference. The R_s_ represents mainly the sheet resistance of the transparent conductive oxide. This is why, all the three R_s_ values obtained in this work are similar [36,37]. The R_ct_ values are nanoporous NiS electrode (2.88 Ω) < flat Pt/FTO electrode (3.03 Ω) < flat NiS/FTO electrode (8.35 Ω), respectively. Obviously, compared with flat NiS/FTO electrode, the R_ct_ value of nanoporous NiS electrode is smaller, and even lower than that of the flat Pt/FTO electrode. This indicates that the morphology plays an important role in adjusting the electrocatalytic activity, and the nanoporous inverse opal structure enables the CE to have a larger specific surface area and more catalytic sites, thereby result a higher electrocatalytic activity. The chemical capacitance of the nanoporous NiS electrode (23.7 × 10^−^^5^ F) is obviously larger than that of the flat Pt/FTO electrode (4.2 × 10^−^^5^ F) and flat NiS/FTO electrode (5.1 × 10^−^^5^ F) (Table S1), demonstrating the higher active surface area and corresponding chemical capacitance charging/discharging characteristics at the porous electrode/electrolyte interface. In addition, the Z_W_ value of nanoporous NiS electrode is the lowest among the three CEs (Table S1), which is due to the diffusion advantage of macroporous structure. Therefore, as an overall impedance effect, the excellent electrocatalytic performance of the nanoporous NiS electrode was obtained.

**Figure 5:**
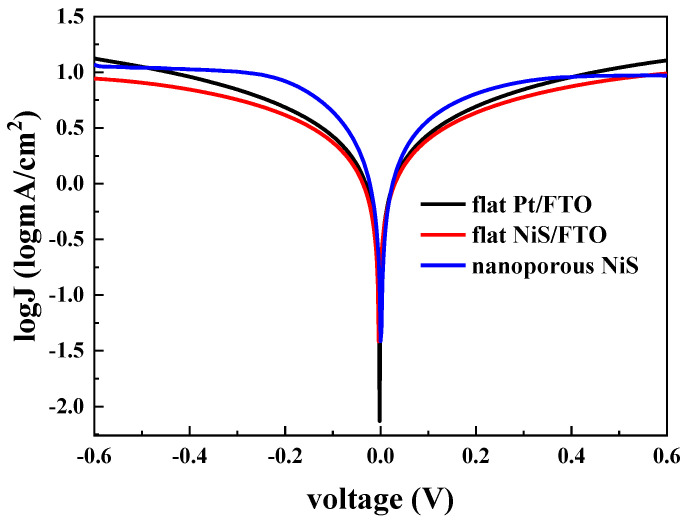
Tafel curves of nanoporous NiS, flat NiS/FTO, and flat Pt/FTO.

The Tafel curves were measured to further evaluate the electrocatalytic activities of the CEs. The tangent of Tafel region intersects with the symmetry line of polarization curve at a certain point, and the corresponding ordinate value of the point is the exchange current density J_0_, which reflects the catalytic ability of the CE to iodine ions in electrolyte [38]. Usually, the larger J_0_ value means the better electrocatalytic activity. As shown in Figure 5, obviously, the J_0_ value of flat NiS/FTO electrode is the lowest, indicating the relatively lowest electrocatalytic performance. Although the Pt/FTO electrode is also a flat electrode, its J_0_ value is higher than flat NiS/FTO due to the extremely excellent electrocatalytic activity of Pt material. However, compared with the traditional CE with flat surface, the nanoporous NiS counter electrode derived of PS template shows excellent electrocatalytic activity, and its J_0_ value is relatively the highest among the three, which indicates that the nanoporous structure has unique advantages compared with the flat NiS/FTO electrode and the Pt/FTO electrode.

**Figure 6:**
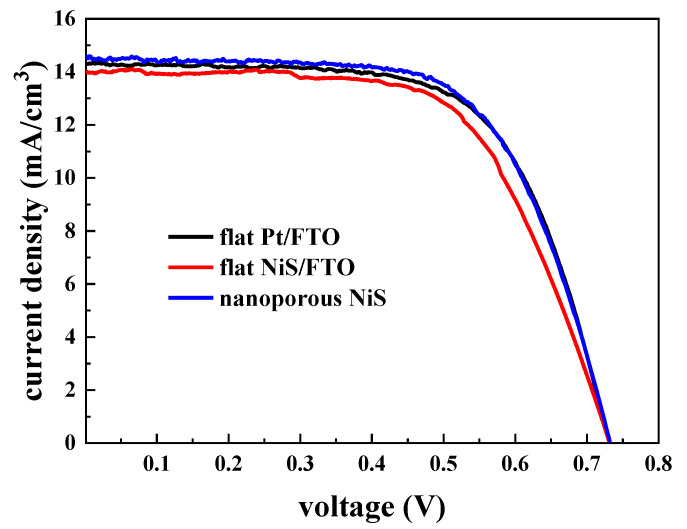
Photocurrent-voltage (J-V) characteristic curves of nanoporous NiS, flat NiS/FTO, and flat Pt/FTO, which were measured under simulated sunlight (100 mW/cm^2^, AM 1.5).

The J-V characteristic curves (Figure 6) can comprehensively reflect the photoelectric conversion performance of CEs in DSSCs [39]. The corresponding parameters of different CEs are summarized in Table 1. Obviously, the performance of flat NiS/FTO (V_OC_ = 0.73 V, J_SC_ = 14.01 mA, FF = 0.62, and PCE = 6.30%) is lower than that of the flat Pt/FTO electrode (V_OC_ = 0.73 V, J_SC_ = 14.29 mA, FF = 0.65, and PCE = 6.69%), which is due to the extremely excellent electrocatalytic performance of Pt material. For the nanoporous NiS electrode, the J_SC_ is 14.51 mA, the filling factor FF is 0.64, and the photovoltaic efficiency (PCE) is 6.77%. Compared with flat NiS/FTO electrode, the V_OC_ has no obvious change, while the J_SC_, FF, and PCE of the nanoporous NiS electrode have obvious improvement, in which the PCE is 7.5% higher than the flat NiS/FTO electrode, which also indicates that the nanoporous NiS electrode has better electrocatalytic performance than the flat NiS/FTO electrode. The better electrocatalytic performance of nanoporous NiS electrode will fasten the regeneration of redox couple and result to a little R_ct_, thereby leading to a small increase in Jsc and PCE. This is consistent with the laws revealed by the EIS plots and the Tafel curves. The photovoltaic parameters of a group of representative samples were shown in Table S2. In order to compare the catalytic performance of the CE used in our work with the most advanced CEs with high photovoltaic efficiency, we listed the corresponding parameters in Table S3. The parameters also illustrate that the comprehensive electrocatalytic performance of nanoporous NiS electrode with inverse opal structure can compete with the traditional Pt/FTO electrode.

## 4. Conclusions

In conclusion, using a template-assisted electrodeposition method, we successfully synthesized nanoporous NiS film with inverse opal structure. The results show that the morphology plays an important role in adjusting the catalytic activity, and the inverse opal structure makes the CE to have a larger specific surface area, thereby result to a higher electrocatalytic activity. Compared with the flat NiS/FTO electrode, this kind of nanoporous NiS electrode exhibited higher electrocatalytic activity. The higher electrocatalytic activity is attributed to the more catalytic sites derived from the larger specific surface area of nanoporous NiS film. Besides, the porous inverse opal structure is also beneficial to the diffusion of I_3_^−^/I^−^ between the electrodes. This study opens up a new experimental exploration for further improving the catalytic activity of NiS and the according photovoltaic efficiency of DSSCs. The PS template-assisted electrodeposition method proposed in this work provides a facile strategy for morphology control and an easy to be realized way to optimize the electrocatalytic performance of metal sulfides. The synthesized nanoporous NiS film can be used as lost-cost Pt-free counter electrode with outstanding electrocatalytic activity to replace the traditional Pt/FTO electrode. It is of great significance to reduce the cost and promote the wide application of DSSCs.

## Figures and Tables

**Table 1:** Photovoltaic parameters of the counter electrodes (CEs) ^a^.

CEs	Rs (Ω/sq)	J_0_ (mA/cm^2^)	R_ct_ (Ω)	J_SC_ (mA/cm^2^)	V_OC_ (V)	FF	PCE (%)
Nanoporous NiS	14.59	1.25	2.88	14.51	0.73	0.64	6.77
Flat NiS/FTO	14.60	1.09	8.35	14.01	0.73	0.62	6.30
Flat Pt/FTO	14.53	1.17	3.03	14.29	0.73	0.65	6.69

^a^ Rs: sheet resistance, J_0_: exchange current density, R_ct_: charge transfer resistance, V_OC_: open circuit voltage, J_SC_: short current density, FF: fill factor, PCE: photovoltaic efficiency.

## Supplementary Materials

The following are available online at https://www.mdpi.com/1996-1944/13/20/4647/s1, Figure S1: Equivalent circuit for fitting electrochemical impedance spectroscopy (EIS) plots, Table S1: Electrochemical impedance spectroscopy (EIS) and Tafel parameters of the counter electrodes (CEs), Table S2: Photovoltaic parameters of a group of representative samples, Table S3: Corresponding parameters of reported CEs with high photovoltaic efficiency.
