# Supplementary material for: Facile Synthesis of Nanoporous NiS Film with Inverse Opal Structure as Efficient Counter Electrode for DSSCs
Source: Materials (Basel). 2020 Oct 18;13(20):4647. doi: 10.3390/ma13204647 (PMC7603251; doi:10.3390/ma13204647)
Supplement: Supplementary file 1 [file materials-13-04647-s001.pdf]

Supplementary Materials

Facile Synthesis of Nanoporous NiS Film with Inverse Opal Structure as Efficient Counter Electrode for DSSCs

Xu Chen [1], Yang Zhang [1,2], Yashuai Pang [1], and Qiwei Jiang [1,2,*]

[1] Department of Physics, School of Physics and Electronic, Henan University, Kaifeng 475004, P. R. China.

[2] Institute of Macro/Nano Photonic Materials and Application, Henan University, Kaifeng 475004, China

* Correspondence: jqw@henu.edu.cn (Q. J.)

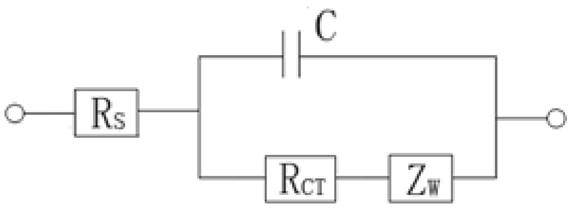

Figure S1 Equivalent circuit for fitting EIS plots. (R_s: sheet resistance, R_{ct}: charge transfer resistance in the electrode/electrolyte interface, C: double layer capacitance, Z_W: Nernst diffusion resistance)

Table S1 EIS and Tafel parameters of the CEs. [a]

CEs	Rs (Ω/sq)	J_0 (mA/cm²)	R_{ct} (Ω)	Z_w (Ω)	C (10^{-5}F)
nanoporous NiS	14.59	1.25	2.88	1.87	23.7
flat NiS/FTO	14.60	1.09	8.35	2.14	5.1
flat Pt/FTO	14.53	1.17	3.03	2.25	4.2

[a] R_s: sheet resistance, J_0: exchange current density, R_{ct}: charge transfer resistance, Z_w: Nernst diffusion impedance, C: double layer capacitance.

Table S2 Photovoltaic parameters of a group of representative samples

CEs		J_{SC} (mA/cm²)	V_{OC} (V)	FF	PCE (%)
nanoporous NiS	Sample A	14.51	0.73	0.64	6.77
	Sample B	14.45	0.73	0.64	6.75
	Sample C	14.41	0.73	0.63	6.68
flat NiS/FTO	Sample A	14.01	0.73	0.62	6.30
	Sample B	13.91	0.73	0.62	6.29
	Sample C	13.94	0.72	0.62	6.22
flat Pt/FTO	Sample A	14.29	0.73	0.65	6.69
	Sample B	14.28	0.73	0.64	6.66
	Sample C	14.06	0.73	0.64	6.57

Table S3 Corresponding parameters of reported CEs with high photovoltaic efficiency

Catalytic material in the CE	PCE (%)	PCE (%) of Pt	Ref.
TiN nanotube arrays	7.73	7.45	[1]
TiN/carbon nanotubes	5.41	5.68	[2]
TiC	6.46	7.23	[3]
MoC	8.34	7.89	[4]
WC	8.18	7.89	[4]
nano-TiC/graphene/PEDOT:PSS	4.5	4.3	[5]
PEDOT:PSS/TiN-NPs	6.67	6.57	[6]
WO_2 nanorod	7.25	7.57	[7]
FeS nanosheets	8.88	7.73	[8]
CoSe	7.39	7.68	[9]
$Co_{0.85}Se$	9.40	8.64	[10]
$Ni_{0.85}Se$	8.32	8.64	[10]
NiS	6.82	7.00	[11]
TiS_2/PEDOT:PSS	7.04	7.65	[12]
nanoporous NiS	6.77	6.69	This work

References:

1. Jiang, Q.; Li, G.; Gao, X. Highly ordered TiN nanotube arrays as counter electrodes for dye-sensitized solar cells, *Chem. Commun.* **2009**, 6720-6722.
2. Li, G.; Wang, F.; Jiang, Q.; Gao, X.; Shen, P. Carbon nanotubes with titanium nitride as a low-cost counter-electrode material for dye-sensitized solar cells, *Angew. Chem. Int. Ed.* **2010**, *49*, 3653-3656.
3. Wang, Y.; Wu, M.; Lin, X.; Hagfeldt, A.; Ma, T. Optimization of the performance of dye‐sensitized solar cells based on Pt‐like TiC counter electrodes, *Eur. J. Inorg. Chem.* **2012**, *2012*, 3557-3561.
4. Wu, M.; Lin, X.; Hagfeldt, A.; Ma, T. Low-Cost Molybdenum Carbide and Tungsten Carbide Counter Electrodes for Dye-Sensitized Solar Cells, *Angew. Chem. Int. Ed.* **2011**, *50*, 3520-3524, S3520/3521-S3520/3527.
5. Peng, Y.; Zhong, J.; Wang, K.; Xue, B.; Cheng, Y. A printable graphene enhanced composite counter electrode for flexible dye-sensitized solar cells, *Nano Energy* **2013**, *2*, 235-240.
6. Yeh, M.; Lin, L.; Lee, C.; Wei, H.; Chen, C.; Wu, C.; Vittal, R.; Ho, K. A composite catalytic film of PEDOT: PSS/TiN–NPs on a flexible counter-electrode substrate for a dye-sensitized solar cell, *J. Mater. Chem.* **2011**, *21*, 19021-19029.

7. Wu, M.; Lin, X.; Hagfeldt, A.; Ma, T. A novel catalyst of WO2 nanorod for the counter electrode of dye-sensitized solar cells, *Chem. Commun.* **2011**, *47*, 4535.
8. Wang, X.; Xie, Y.; Bateer, B.; Pan, K.; Zhou, Y.; Zhang, Y.; Wang, G.; Zhou, W.; Fu, H. Hexagonal FeS nanosheets with high-energy (001) facets: Counter electrode materials superior to platinum for dye-sensitized solar cells, *Nano Res.* **2016**, *9*, 2862-2874.
9. Murugadoss, V.; Wang, N.; Tadakamalla, S.; Wang, B.; Guo, Z.; Angaiah, S. In situ grown cobalt selenide/graphene nanocomposite counter electrodes for enhanced dye-sensitized solar cell performance, *J. Mater. Chem. A* **2017**, *5*, 14583-14594.
10. Gong, F.; Wang, H.; Xu, X.; Zhou, G.; Wang, Z.-S. In situ growth of Co0. 85Se and Ni0. 85Se on conductive substrates as high-performance counter electrodes for dye-sensitized solar cells, *J. Am. Chem. Soc.* **2012**, *134*, 10953-10958.
11. Sun, H.; Qin, D.; Huang, S.; Guo, X.; Li, D.; Luo, Y.; Meng, Q. Dye-sensitized solar cells with NiS counter electrodes electrodeposited by a potential reversal technique, *Energy Environ. Sci.* **2011**, *4*, 2630-2637.
12. Li, C.; Lee, C.; Li, Y.; Yeh, M.; Ho, K. A composite film of TiS2/PEDOT:PSS as the electrocatalyst for the counter electrode in dye-sensitized solar cells, *Journal of Materials Chemistry A* **2013**, *1*, 14888-14896.
